# Long-term impact of the COVID-19 pandemic on childhood vaccination coverage in Quebec, Canada: A cohort study from the Canadian immunization research network

**DOI:** 10.1016/j.pmedr.2026.103425

**Published:** 2026-02-20

**Authors:** Marilou Kiely, Iulia Gabriela Ionescu, Mourad Dahhou, Ève Dubé, Chantal Sauvageau, Laura Reifferscheid, Shannon E. MacDonald

**Affiliations:** a Université Laval, 1050 Av. de la Médecine, G1V 0A6 Québec, Canada; b Centre de recherche du CHU de Québec – Université Laval, 2400 Av. D'Estimauville, G1E 6W2 Québec, Canada; c Institut national de santé publique du Québec, 945 Av. Wolfe, G1V 5B3 Québec, Canada; d University of Alberta, 116 St & 85 Av., AB T6G 2R3 Edmonton, Canada

**Keywords:** Vaccination coverage, Public health, Vaccine timeliness, COVID-19, Vaccine-preventable diseases

## Abstract

**Objective:**

The COVID-19 pandemic disrupted routine childhood vaccination globally, increasing the risk of resurgences of vaccine-preventable diseases. This study assessed the long-term impact of the pandemic on vaccination coverage and timeliness for routine childhood immunizations for children born in 2017–2023.

**Methods:**

We conducted a retrospective cohort study in Quebec, Canada, using data from the Quebec Immunization Registry. We compared age-appropriate vaccination coverage by 3, 5, 13 and 19 months of age and the cumulative days undervaccinated during the first 24 months of life, from 2019 to 2023.

**Results:**

There was no decrease in vaccination coverage by 3 and 5 months of age in 2023 compared to 2019 for DTaP, Hepatitis B, rotavirus and pneumococcal conjugate vaccines. We observed a 0.8 percentage point decline (95% confidence intervals (CI):-1.2,-0.4) for ≥1 dose of the measles vaccine by 13 months of age, and a 1.0 percentage point decline (95%CI:-1.5,-0.06) for ≥2 doses by 19 months of age in 2023 compared to 2019. Overall, the mean number of days undervaccinated decreased across birth cohorts from 158.8 in 2019 to 133.8 in 2023.

**Conclusion:**

Delays in the administration of the measles vaccine remain concerning. Sustained efforts are needed to maintain high vaccination coverage and prevent outbreaks of vaccine-preventable diseases.

## Highlights

• This study evaluates the long-term impact of COVID-19 on childhood vaccination
• No decrease in coverage for vaccines given before 12 months in 2023 versus 2019
• Slight decline in measles vaccine coverage in 2023 versus 2019
• Mean number of days undervaccinated decreased across cohorts from 2019 to 2023

## 1 Introduction

Routine childhood immunization is crucial to prevent and control vaccine-preventable diseases (VPDs) and their complications (Bos and Baston, 2020). The COVID-19 pandemic disrupted routine vaccination globally, leading to declines in vaccination coverage and increasing the risk of resurgence of VPDs, such as measles (World Health Organization, 2020a; World Health Organization, 2020b). Additionally, lockdowns and fear of contracting the virus impacted the use of health services and further contributed to missed or delayed childhood vaccinations (Public Health Agency of Canada, 2020; Ji et al., 2022; Evans et al., 2023).

In Canada, several studies documented declines in vaccination coverage during the first months of the pandemic (Ji et al., 2022; Dong et al., 2022; MacDonald et al., 2022; Sell et al., 2021). A recent analysis conducted by the Public Health Agency of Canada assessed changes in vaccination coverage at ages 2 and 7 years before, during, and after the pandemic in four provinces. Findings showed a decline in coverage in 2023 compared to 2019 for the diphtheria, tetanus, and acellular pertussis (DTaP) and the measles, mumps, and rubella (MMR) vaccines. In 2-year-olds, coverage for both vaccines decreased by 7 percentage points (Jeevakanthan et al., 2025). Many countries, including Canada, have recently experienced large measles outbreaks (World Health Organization, 2025; Government of Canada, 2025).

In Quebec, Canada's second most populous province (population of 9.1 million), the routine childhood immunization schedule for children born from June 2019 onwards includes vaccination visits at 2, 4, 12, and 18 months of age, covering 14 vaccine antigens (12 doses). The pandemic initially led to a decrease in vaccination coverage among children aged <2 years in Quebec, especially for vaccines scheduled at 18 months of age (i.e., measles-containing vaccine and DTaP-containing vaccine) (Kiely et al., 2022). Vaccination coverage improved following recommendations issued in May 2020 to resume all routine vaccinations for infants and preschoolers (Kiely et al., 2022; Comité sur l'immunisation du Québec, 2019). Despite catch-up efforts, data from a provincial survey (2021), showed a decrease of 3.1 percentage points in coverage for the complete routine immunization schedule by 24 months of age compared to 2019 (Kiely et al., 2021). It remains uncertain whether the decline in vaccination coverage has persisted over time. In addition, recovery to pre-pandemic levels may have been affected by a decrease in perceived vaccine importance and an increase in vaccine hesitancy for routine childhood vaccines (UNICEF, 2023; Lopes et al., 2022; Vashist et al., 2024).

Vaccination coverage data beyond the initial months of the pandemic are limited and could help clarify current vulnerability to VPDs and inform future interventions. Documenting vaccination delays is also essential to understand post-pandemic immunization schedule adherence (Newcomer et al., 2023). The aim of this study was therefore to assess the long-term impact of the COVID-19 pandemic on age-appropriate vaccination coverage for routine early childhood immunizations (administered between 0 and 24 months of age) for children born 2017–2023, providing evidence on whether the early declines in coverage persisted or recovered over time. We also estimated the number of days children spent undervaccinated before the age of 2 years.

## 2 Methods

### 2.1 Study design and population

We conducted a retrospective cohort study in Quebec, Canada, to assess vaccination coverage and delays between January 1, 2019, to December 31, 2023. We used data from the Quebec Immunization Registry, a population-based registry that includes both vaccinated and unvaccinated individuals, to identify targeted children and obtain immunization data. The Registry contains information on vaccine product, number of doses and vaccination dates for all vaccines administered in Quebec as well as sociodemographic data regularly updated by the Quebec provincial public health insurance database. All facilities providing vaccines are required to record all administered vaccines in the registry. For each year of the study period, more than 97% of doses administered to children aged 0–3 years were recorded within 90 days of their administration date. Only children linked with the health insurance database were included (±96% of all children). Children who passed away (±0.1%) or with no recorded address in Quebec (±1%) were excluded. Data were extracted on October 30, 2024.

### 2.2 Outcomes measured

#### 2.2.1 Age-appropriate vaccination coverage

Birth cohorts included in our study had different vaccination schedules (Table 1). For children born from June 2019 onwards, vaccines routinely recommended in early childhood include 3 doses of the combination vaccine against diphtheria, tetanus toxoids and acellular pertussis (DTaP), poliovirus (IPV), *Haemophilus influenzae* type b (Hib), including 2 doses of hepatitis B; 3 doses of the pneumococcal conjugate vaccine (PCV); 2 doses of the rotavirus vaccine; and 2 doses of the combined measles, mumps, rubella, varicella vaccine (MMRV).

**Table 1:** Quebec routine vaccination schedule up to 24 months of age for children born from June 2017 to September 2023.

Vaccination visit (target age)	Born before June 1, 2019	Born on or after June 1, 2019
2 months	• DTaP-HB-IPV-Hib • Rotavirus • PCV	• DTaP-HB-IPV-Hib • Rotavirus • PCV
4 months	• DTaP-HB-IPV-Hib • Rotavirus • PCV	• DTaP-HB-IPV-Hib • Rotavirus • PCV
6 months	• DTaP-IPV-Hib	–
12 months	• PCV • MMR±V^a^ • Men-C-C	• DTaP-IPV-Hib • PCV • MMRV
18 months	• DTaP-HB-IPV-Hib • MMRV	• MMRV • Men-C-C • HA-HB

DTaP-HB-IPV-Hib: diphtheria, tetanus, acellular pertussis, hepatitis B, polio, and *Haemophilus influenzae* type b vaccine; DTaP-IPV-Hib: diphtheria, tetanus, acellular pertussis, polio, and *Haemophilus influenzae* type b vaccine; HA-HB: combined hepatitis A and B vaccine; Men-C-C: meningococcal conjugate serogroup C vaccine; MMR ± V: measles, mumps, rubella and varicella vaccine; N/A: Not applicable; PCV: pneumococcal conjugate vaccine;

a For this vaccination visit, the combined measles, mumps, rubella and varicella vaccine (MMRV) was offered only to children born on or after June 1, 2018.

We estimated age-appropriate vaccination coverage for each recommended vaccination visit (i.e., 2, 4, 12, and 18 months of age) annually. Vaccination coverage was calculated using the proportion of adequately vaccinated children among children who reached each target age each year from 2019 to 2023 (i.e., 3, 5, 13 and 19 months of age). To be considered adequately vaccinated, children must have received the recommended vaccines within one month of the targeted age of vaccination (i.e., the day before reaching 3, 5, 13 and 19 months of age) (Health Canada, 2004). Analysis was limited to routine vaccine doses offered at the same vaccination visit to children born before June 2019 and those born on or after June 2019 (Table 1). To assess the impact of the pandemic on vaccination, we compared vaccination coverage at each target age in 2020, 2021, 2022, and 2023 with that of 2019 (pre-pandemic period). We also calculated the difference in cumulative vaccination coverage by 13 months versus 15 months of age and by 19 months versus 24 months of age for the measles vaccine, for each year, to assess whether allowing more time for the same cohort of children substantially increased vaccination coverage.

#### 2.2.2 Days Undervaccinated

To further assess vaccination delays, we estimated the number of days for which each child was considered undervaccinated for each vaccine dose (Luman et al., 2005). Depending on the vaccine and recommended dose, delay counts started on the day the child reached 3, 5, 13 or 19 months of age and accumulated until the child received their vaccine or reached 24 months of age, except for the rotavirus vaccine, for which all doses must be administered before 8 months of age and no catch-up is possible beyond this age (Supplementary Table 1). Children who received doses after these cutoffs were considered unvaccinated. Only children who were at least 24 months old at the end of the study period for each year were included in this analysis (i.e., those born from June 2017 to May 2022 and evaluated for vaccination coverage by 19 months each year from 2019 to 2023). Days undervaccinated for each vaccine were estimated by summing all days a child was undervaccinated for 1 or more doses of each vaccine. We also computed days undervaccinated for all vaccines by summing all days undervaccinated for at least 1 dose of any vaccines. Overlapping days during undervaccinated periods (i.e., being undervaccinated for ≥1 vaccine dose at the same time) were only counted once. Finally, we estimated the total average number of days undervaccinated (ADU), by dividing the total number of days undervaccinated for each vaccine by the number of recommended vaccines included in the analysis (Glanz et al., 2013).

### 2.3 Statistical analysis

For all analyses, doses administered before the minimum age for vaccination, and doses that did not meet the minimum interval between doses within a series, were considered invalid and were not counted as received. Overall, 0.003% of included children had inconsistencies between date of birth and vaccination date. Age-appropriate vaccination coverage and corresponding 95% confidence intervals (CI) for each vaccine and dose were estimated using the exact (Clopper-Pearson) method for binomial proportions for each year from 2019 to 2023. We assessed trends in age-appropriate vaccination coverage for each vaccine across years using the Cochran-Armitage trend test. We then calculated differences in vaccination coverage at each targeted age for 2020–2023 compared with 2019, along with the corresponding 95% CI for the differences using the same method to evaluate whether pandemic vaccination coverage declines recovered over time. For analyses regarding vaccination delays, the mean number of days undervaccinated with the standard deviation (SD) were calculated by vaccine and dose from 2019 to 2023. We also reported the mean of the ADU with the SD. Vaccination coverage by age in months was also calculated according to the Kaplan-Meier method, representing the cumulative probability of being vaccinated at age *t* among the children at the same age by 24 months of age (Siedler et al., 2002). Finally, sensitivity analyses were conducted (a) excluding the two northern health regions of Quebec, since greater delays in data entry were expected in these regions, and (b) excluding the rotavirus vaccine from the estimation of ADU. All analyses were performed using SAS software version 9.4.

### 2.4 Ethical statement

Ethical approval for this study was obtained from the Research Ethics Committee of the Centre Hospitalier Universitaire (CHU) de Québec - Université Laval (#2024–7112). We followed the Strengthening the Reporting of Observational Studies in Epidemiology (STROBE) guidelines (Von Elm et al., 2008).

## 3 Results

### 3.1 Number of children included in the analysis

Cohorts for the vaccination coverage evaluation by 3, 5, 13 and 19 months from 2019 to 2023 totalled 436,180, 439241, 447670 and 453,855 children, respectively. The calculation of days undervaccinated included only children with complete follow-up through 24 months of age for each year (i.e., 453855 children) (Table 2).

**Table 2:** Cohorts of children included in the analysis by vaccination coverage measure and year, Quebec 2019–2023.

Vaccination coverage measures	Year	Birth cohorts included	Number of children included^a^	Number of children included (excluding the two northern health regions of Quebec)^a^
by 3 months of age	2019	Oct 1, 2018 - Sept 30, 2019	92,168	91,606
	2020	Oct1, 2019 – Sept 30, 2020	88,513	87,965
	2021	Oct 1, 2020 – Sept 30, 2021	89,432	88,782
	2022	Oct 1, 2021 – Sept 30, 2022	86,149	85,546
	2023	Oct 1, 2022 – Sept 30, 2023	79,918	79,463
by 5 months of age	2019	Aug 1, 2018 – Jul 31, 2019	92,465	91,904
	2020	Aug 1, 2019 – Jul 31, 2020	89,812	89,261
	2021	Aug 1, 2020 – Jul 31, 2021	88,203	87,569
	2022	Aug 1, 2021- Jul 31, 2022	87,638	87,026
	2023	Aug 1, 2022 – Jul 31, 2023	81,123	80,623
by 13 months of age	2019	Dec 1, 2017 – Nov 30, 2018	93,024	92,497
	2020	Dec 1, 2018 – Nov 30, 2019	91,889	91,338
	2021	Dec 1, 2019 – Nov 30, 2020	87,105	86,526
	2022	Dec 1, 2020 – Nov 30, 2021	90,778	90,126
	2023	Dec 1, 2021 – Nov 30, 2022	84,874	84,295
by 19 months of age^b^	2019	June 1, 2017 - May 31, 2018	93,385	92,697
	2020	June 1, 2018 – May 31,2019	92,787	92,116
	2021	June 1, 2019 – May 31, 2020	90,784	90,102
	2022	June 1, 2020 – May 31, 2021	87,038	86,293
	2023	June 1, 2021 - May 31, 2022	89,861	89,137

a The denominators included children who reached each target age each year from 2019 to 2023 (i.e., 3, 5, 13 and 19 months of age).

b Only those children have been included in the calculation of days undervaccinated.

### 3.2 Age-appropriate vaccination coverage

**Fig. 1:**
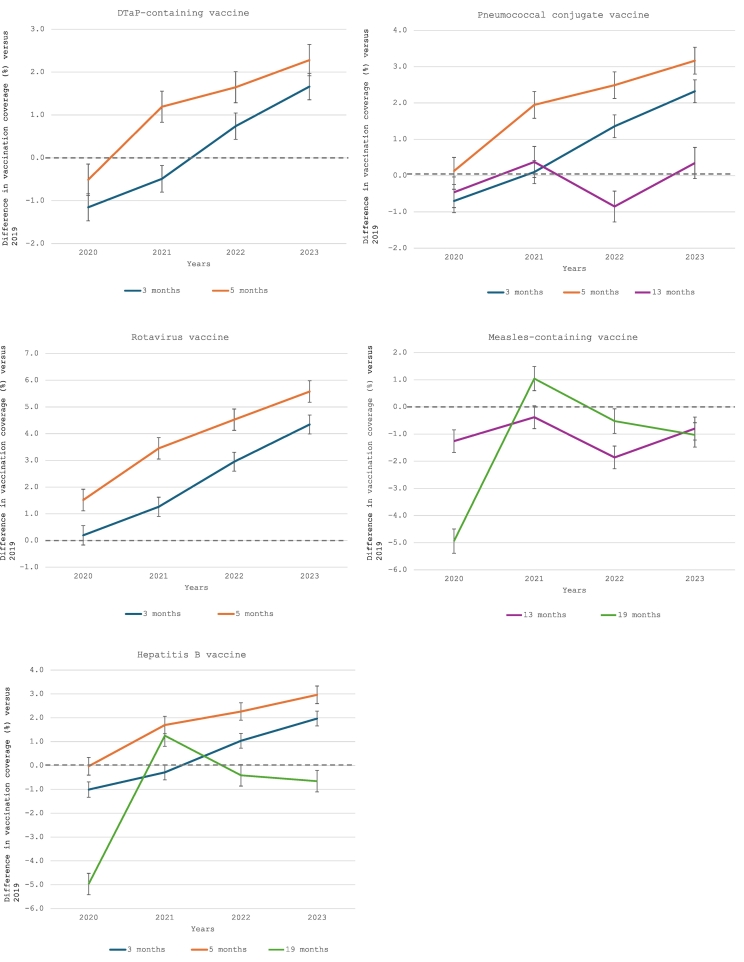
**Difference in age-appropriate vaccination coverage among Quebec children for vaccines recommended at the 2-, 4-, 12- and 18-month vaccination visits, for each year from 2020 to 2023 compared to pre-pandemic period (2019).** DTaP-containing vaccine: all vaccines containing diphtheria, tetanus, acellular pertussis antigens (e.g. DTaP-HB-IPV, DTaP-IPV-Hib); Measles-containing vaccine: all vaccines containing measles antigen (e.g. MMR, MMRV); The horizontal line at 0 indicates no difference in vaccination coverage compared with the 2019 estimates. The vertical bars represent the 95% confidence intervals (95% CI) for the difference in vaccination coverage.

Vaccination coverage by 3 months of age for DTaP and PCV vaccines was higher in 2022 and 2023 compared to 2019, as the differences in coverage was >0 for both years (Fig. 1). Similar trends were observed for vaccination coverage by 5 months of age with an increase in coverage starting in 2021. Vaccination coverage for the rotavirus vaccine did not experience a drop in 2020 and has increased steadily each year since 2019. Hepatits B vaccine findings were similar to those for the DTaP vaccine, as the combined INFANRIX hexa® vaccine was primarily used.

**Fig. 2:**
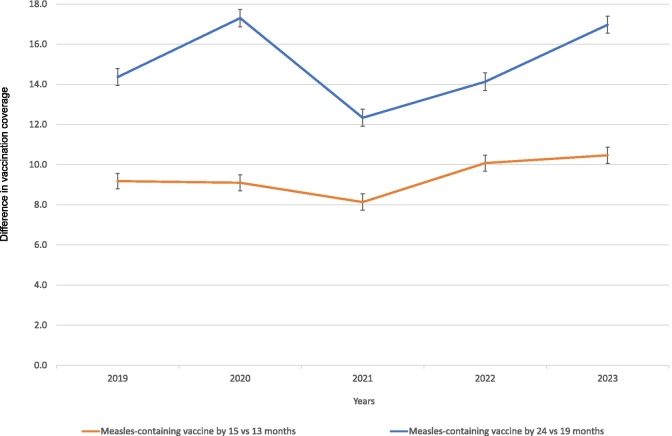
**Difference in vaccination coverage among Quebec children for the measles-containing vaccine by 15 and 24 months of age compared to age-appropriate vaccination for the 12- and 18-month vaccination visits (i.e. by age 13 months and 19 months, respectively), for each year from 2019 to 2023.** Measles-containing vaccine: all vaccines containing measles antigen (e.g. MMR, MMRV). The vertical bars represent the 95% confidence intervals (95% CI) for the difference in vaccination coverage.

Vaccination coverage by 13 months of age was slightly higher for the PCV vaccine in 2023 compared to 2019, whereas vaccination coverage for the measles vaccine was higher in 2019 than in subsequent years (−0.8 percentage points (95% CI:-1.2,-0.4) in 2023 versus 2019) (Fig. 1). After a significant drop in measles vaccination coverage by 19 months of age in 2020, coverage increased in 2021; however, coverage in 2022 and 2023 remained slightly lower than pre-pandemic level (−1.0 percentage point (95% CI:-1.5,-0.6) in 2023 versus 2019). Similar trends were observed for Hepatitis B vaccination coverage by 19 months of age. Finally, the difference in cumulative measles vaccination coverage between 15 and 13 months (10.5 percentage points in 2023 versus 9.2 percentage points in 2019) and between 24 and 19 months (17.0 percentage points in 2023 versus 14.4 percentage points in 2019) was greater in 2023 than in 2019, as more children had received their delayed first and second doses by 15 and 24 months of age, respectively, in 2023 (Fig. 2). Supplementary Table 2 shows all vaccination coverage estimates and 95% CIs for analysed vaccines as well as the results of trend analyses across years (all *p*-values for trends <0.05 except for PCV by 13 months of age). Sensitivity analyses excluding the two northern health regions produced similar conclusions, with marginally smaller differences between 2023 and 2019 for the rotavirus vaccine by 3 months of age and both doses of the measles vaccine (Supplementary Table 3).

### 3.3 Vaccination delays

Overall, the mean days undervaccinated decreased across birth cohorts who had their 18-month vaccination visit between 2019 and 2023 (Table 3). Indeed, the mean days undervaccinated was 133.8 (SD: 0.7) in 2023 compared to 158.8 (SD: 0.8) in 2019. Similar results were observed when examining the average number of days undervaccinated. In 2023, the mean was 67.7 days (SD: 0.2) compared to 75.0 (SD: 0.2) in 2019. For all individual vaccines, except for the measles vaccine, the proportion of children with a vaccine delay was lower in 2023 compared to 2019. For the measles vaccine, 46.2% of children experienced a delay in 2023 versus 44.5% in 2019. However, the number of days undervaccinated for measles was lower in 2023 (78.5, SD: 0.4) compared to 2019 (80.6, SD: 0.4), as more children had completed their vaccine series in 2023 (Supplementary Table 2 and Supplementary Fig. 1). Overall conclusions did not change after excluding the rotavirus vaccine; however, the mean days undervaccinated and the average number of days undervaccinated were lower each year (Table 3 and Supplementary Table 4).

**Table 3:** Days undervaccinated for vaccines included in the analysis among Quebec children born from June 2017 to May 2023, 2019–2023.

	Birth Cohort (No. combined = 453,855)^a^				
	Children born from June 2017 to May 2018	Children born from June 2018 to May 2019	Children born from June 2019 to May 2020	Children born from June 2020 to May 2021	Children born from June 2021 to May 2022
	18-month visit in 2019	18-month visit in 2020	18-month visit in 2021	18-month visit in 2022	18-month visit in 2023
Children included in the analysis	93,385	92,787	90,784	87,038	89,861
No. days undervaccinated, Mean (SD)					
Pneumococcal conjugate vaccine					
Children with vaccine delay^b^, n (%)	35,670 (38.2)	33,786 (36.4)	31,294 (34.5)	30,632 (35.2)	31,483 (35.0)
Dose 1	77.4 (0.7)	68.7 (0.6)	66.4 (0.6)	68.3 (0.7)	58.7 (0.6)
Dose 2	82.7 (0.6)	74.1 (0.6)	74.9 (0.6)	75.4 (0.6)	67.2 (0.6)
Dose 3	73.6 (0.4)	69.6 (0.4)	69.1 (0.4)	70.4 (0.4)	64.5 (0.4)
Total^c^	82.0 (0.5)	77.6 (0.5)	78.3 (0.5)	79.0 (0.5)	73.7 (0.5)
Hepatitis B					
Children with vaccine delay^b^ n (%)	41,461 (44.4)	44,997 (48.5)	38,690 (42.6)	37,444 (43.0)	39,246 (43.7)
Dose 1	77.1 (0.7)	69.3 (0.6)	68.2 (0.6)	70.2 (0.7)	60.0 (0.6)
Dose 2	85.2 (0.7)	77.6 (0.6)	78.8 (0.6)	79.4 (0.7)	70.7 (0.6)
Dose 3	45.5 (0.2)	50.5 (0.2)	45.8 (0.2)	46.4 (0.2)	44.0 (0.2)
Total^c^	102.1 (0.6)	102.3 (0.6)	98.7 (0.6)	99.6 (0.6)	92.1 (0.6)
Rotavirus					
Children with vaccine delay^b^ n (%)	28,532 (30.6)	26,285 (28.3)	23,967 (26.4)	21,537 (24.7)	21,368 (23.8)
Dose 1	30.4 (0.2)	27.7 (0.2)	24.9 (0.2)	25.1 (0.2)	21.9 (0.2)
Dose 2	22.5 (0.1)	20.7 (0.1)	19.4 (0.1)	18.8 (0.1)	17.2 (0.1)
Total^c^, ^d^	35.2 (0.2)	32.4 (0.2)	29.9 (0.2)	29.5 (0.2)	26.6 (0.2)
Measles-containing vaccine^e^					
Children with vaccine delay^b^ n (%)	41,531 (44.5)	45,738 (49.3)	38,614 (42.5)	39,316 (45.2)	41,529 (46.2)
Dose 1	60.2 (0.4)	59.0 (0.4)	60.6 (0.4)	62.2 (0.4)	57.6 (0.4)
Dose 2	43.1 (0.2)	48.2 (0.2)	44.0 (0.2)	44.5 (0.2)	42.6 (0.2)
Total^c^	80.6 (0.4)	84.6 (0.4)	80.8 (0.4)	82.6 (0.4)	78.5 (0.4)
All vaccines^f^	158.8 (0.8)	154.3 (0.7)	144.6 (0.7)	144.8 (0.8)	133.8 (0.7)
All vaccines (without rotavirus)	148.2 (0.8)	144.2 (0.7)	136.7 (0.7)	137.3 (0.8)	127.2 (0.7)
Average no of days undervaccinated^g^	75.0 (0.2)	74.2 (0.2)	71.9 (0.2)	72.6 (0.2)	67.7 (0.2)
Average no of days undervaccinated (without rotavirus)	66.2 (0.3)	66.1 (0.3)	64.5 (0.3)	65.3 (0.3)	61.1 (0.3)

SD: Standard deviation.

a Cohorts were selected based on vaccination visits scheduled each year from 2019 to 2023. Only the cohorts selected for the 18-month vaccination visit had complete follow-up to 24 months and were included in the analysis of vaccination delays.

b At least one dose that is delayed.

c Days undervaccinated for each specific vaccine were calculated by summing all days during which the child was undervaccinated for at least one dose of that vaccine. Overlapping days are only counted once. For instance, if a child is considered undervaccinated for dose 1 of the rota vaccine on days 90 to 180 (received the dose at 6 months) and for dose 2 on days 151 to 210 (received the dose at 7 months), the overlapping days from 151 to 180 were only counted once and the child will be considered undervaccinated for a total of 121 days (from 90 days to 210 days).

d Delay count for rotavirus vaccine assessed up to 8 months of age (242–245 days). All doses were considered invalid if the first dose was administered after 20 weeks. Any dose given after 242–245 days was deemed invalid.

e Measles-containing vaccine: all vaccines containing measles antigen (e.g. MMR; MMR ± V).

f Days undervaccinated for all vaccines were calculated by summing all days during which the child was undervaccinated for at least one dose of any vaccines included in the analysis. Overlapping days are only counted once, using the same approach as presented in note C.

g Calculated as the total number of days undervaccinated summed across all vaccine series divided by the number of vaccines recommended (no = 4).

## 4 Discussion

Among children born from 2017 to 2023, vaccination coverage by 3 and 5 months of age was higher in 2023 compared to 2019 for all vaccines. Similar results were observed for the PCV coverage by 13 months of age. However, a slight decrease in coverage for the first dose of the measles vaccine was noted in 2023 compared to 2019 (−0.8 percentage point, corresponding to approximately 680 fewer children in our cohort having received the first dose by 13 months of age). Vaccination coverage for the 18-month vaccination visit, which was the most impacted during the first months of the pandemic, was also slightly lower in 2023 compared to 2019 (−1.0 percentage point). This corresponds to approximately 900 fewer children in our cohort having received the 2 doses of measles by 19 months of age. Our findings also indicate that more children were caught up on their measles vaccinations between 13 and 15 months (first dose) and 19–24 months (second dose) in 2023 compared to 2019. However, vaccination coverage by 24 months of age did not reach the 95% threshold required for herd immunity. Looking further into vaccine timeliness, we found that the mean number of days undervaccinated for all vaccines, as well as for each individual vaccine included in this analysis, was lower in 2023 compared to 2019. However, the proportion of children experiencing a vaccine delay for any dose of measles vaccine was higher in 2023 compared to 2019.

Our results differed from those published by Jeevakanthan et al., who reported a 7 percentage point decrease for MMR vaccine and a 7.8 percentage point decline for DTaP vaccine in 2023 compared to 2019 among 2-year-old children (Jeevakanthan et al., 2025). This Canadian study included data from four provinces, excluding children from Quebec, and used a different method than ours to estimate vaccination coverage (e.g. the denominators). A U.S. study using data from the National Immunization Survey-Child reported lower vaccination coverage for most vaccines among children born in 2020 and 2021 compared to those born in 2018 and 2019 (Hill et al., 2024). The decrease ranged from 1.3 to 3.2 percentage points depending on vaccines. They found a 1.7 percentage point decline for ≥1 dose of the MMR vaccine. These results are generally consistent with ours, as we also observed a decrease in vaccination coverage for some vaccines in 2022 (birth cohorts December 2020–November 2021) compared to 2019 (birth cohorts December 2017–November 2018). Another U.S. study conducted in 9 jurisdictions using Immunization Information Systems found a 10.4 percentage point decrease in the complete vaccine series by 2 years of age in children born in 2020 compared to those born in 2016 (Treharne et al., 2024). Similarly, we also found a decrease in DTaP vaccination coverage by 3 months of age in 2020–2021 (birth cohorts October 2019–September 2021). They also reported a 14.9 percentage point decline in coverage at 2 years of age for ≥1 dose of the MMR vaccine between the 2016 and 2020 birth cohorts, which was much greater than what we observed in this study among birth cohorts from December 2020 to November 2021. Compared to both U.S. studies, our evaluation included a longer follow-up.

Recently, the province of Quebec experienced large outbreaks of measles and pertussis (Comité sur l'immunisation du Québec, 2025). Sustained efforts were made to update childhood vaccinations and ensure all vaccination data were entered into the registry. This could have contributed to the increase in vaccination coverage for the DTaP vaccine in 2023, as well as the smaller decrease for the measles vaccine observed in our study compared with other studies. Despite efforts, a potential increase in vaccine hesitancy for routine childhood vaccines post-pandemic may have contributed to lower vaccination coverage and greater vaccine delays observed for the measles vaccine in 2023 (Humble et al., 2023; Public Health Agency of Canada, 2024).

This study has several limitations. First, we were unable to assess coverage and timeliness for all vaccines in the complete series due to vaccination schedule changes during the study period and since not all birth cohorts had reached 24 months of age by the end of follow-up. Second, despite the implementation of the registry since 2014 and data entry requirements, underestimation of vaccination coverage and overestimation of the number of days undervaccinated is still possible (vaccines administered but not entered in the registry). However, as more than 97% of doses administered were recorded within 90 days of their administration date each year and given that the data extraction occurred more than 6 months after the last scheduled vaccination visit, the impact on our evaluation of trends is expected to be minimal. Third, we presented unadjusted estimates to evaluate overall trends in vaccination coverage. As we used a population-based administrative database capturing almost the entire target population, we expect changes in the composition of the population to be minimal and that unadjusted estimates accurately reflect population-level trends. Fourth, as previously noted, recent measles outbreaks may have improved the completeness of vaccination data entry, which could have potentially contributed to the observed increase in vaccination coverage in 2023 for some vaccines. Finally, this study did not capture disparities in vaccination coverage by sociodemographic characteristics or at the regional level. Despite these limitations, our study provides a comprehensive evaluation of the pandemic's long-term impact on routine immunization in children, using a population-based registry that captures data from nearly all children in Quebec. Additionally, data in the registry are recorded by health care providers administering the vaccines, which enhances the validity of the measurement of outcomes.

In the context of resurgence of certain VPDs, continuous monitoring of vaccination coverage and vaccine delays in the post-pandemic era is essential to rapidly detect any changes in vaccine acceptance or access barriers. Monitoring trust in vaccines and lasting consequences of the pandemic on parents' attitudes regarding routine childhood vaccines is also required to inform interventions to enhance acceptance. In addition, future work could include stratified analyses to assess disparities in vaccination coverage by sociodemographic characteristics and region. Our study shows that immunization registries are useful tools to identify children who missed vaccinations during the pandemic and disparities in vaccination coverage that may have widened in the post-pandemic period. Findings of such analysis are crucial to inform public health activities to improve vaccination coverage (Michels et al., 2024).

## 5 Conclusion

Higher vaccination coverage observed in this study for some vaccines in 2023 compared to 2019 is reassuring; however, delays in the administration of the measles vaccine and coverage by 24 months of age below the 95% threshold required for herd immunity remain concerning. Sustained, evidence-based efforts are also needed to increase and maintain high routine vaccination coverage and protect children against VPDs. Immunization registry data are essential to monitor trends, identify gaps, and guide timely interventions.

## CRediT authorship contribution statement

**Marilou Kiely:** Writing – review & editing, Writing – original draft, Visualization, Validation, Resources, Project administration, Methodology, Data curation, Conceptualization. **Iulia Gabriela Ionescu:** Writing – review & editing, Writing – original draft, Visualization, Methodology, Formal analysis, Data curation, Conceptualization. **Mourad Dahhou:** Writing – review & editing, Visualization, Validation, Methodology, Formal analysis, Data curation. **Ève Dubé:** Writing – review & editing, Visualization, Validation, Methodology, Conceptualization. **Chantal Sauvageau:** Writing – review & editing, Visualization, Validation, Methodology, Conceptualization. **Laura Reifferscheid:** Writing – review & editing, Visualization, Validation, Methodology, Conceptualization. **Shannon E. MacDonald:** Writing – review & editing, Visualization, Validation, Methodology, Funding acquisition, Conceptualization.

## Funding statement

This study was supported by the 10.13039/501100012395Canadian Immunization Research Network.

## Declaration of competing interest

The authors declare the following financial interests/personal relationships which may be considered as potential competing interests: Marilou Kiely reports financial support was provided by Canadian Immunization Research Network. Marilou Kiely reports a relationship with Quebec Immunization Committee that includes: board membership. Eve Dube reports a relationship with National Advisory Committee on Immunization that includes: board membership. Chantal Sauvageau reports a relationship with Quebec Immunization Committee that includes: board membership. If there are other authors, they declare that they have no known competing financial interests or personal relationships that could have appeared to influence the work reported in this paper.

## Data Availability

The authors do not have permission to share data.

## Appendix A Supplementary data

Supplementary material 1 Image 1

Supplementary material 2 Image 2
