# Supplementary material for: Long-term impact of the COVID-19 pandemic on childhood vaccination coverage in Quebec, Canada: A cohort study from the Canadian immunization research network
Source: Prev Med Rep. 2026 Feb 20;64:103425. doi: 10.1016/j.pmedr.2026.103425 (PMC12955240; doi:10.1016/j.pmedr.2026.103425)
Supplement: Supplementary file 1 — Supplementary material 1 [file mmc1.docx]

**Supplementary material**

**Supplementary Table 1** Recommended, minimum ages and delay count for routine vaccinations included in this analysis among Quebec children born from June 2017 to May 2022

| **Vaccines and doses** | **Recommended age for routine vaccination** | **Minimum age for vaccination** | **Minimum interval between doses** | **Age when delay count initiated (age in days)^a^** | **Maximum number of days overdue at 24 month^b^** |  |
| --- | --- | --- | --- | --- | --- | --- |
| Hepatitis B^c^ |  |  |  |  |  |  |
| Dose 1 | 2 months | 6 weeks | - | 3 months (90-93) | 641 |  |
| Dose 2 | 4 months | 10 weeks | 4 weeks | 5 months (151-154) | 580 |  |
| Dose 3 | 18 months | 12 months | 4 weeks | 19 months (578-581) | 153 |  |
| Total hepatitis B | | | | | 641 (730 minus 89) | |
| Pneumococcal conjugate vaccine^d^ |  |  |  |  |  |  |
| Dose 1 | 2 months | 6 weeks | - | 3 months (90-93) | 641 |  |
| Dose 2 | 4 months | 10 weeks | 4 weeks | 5 months (151-154) | 580 |  |
| Dose 3 | 12 months | 12 months | 8 weeks | 13 months (394-397) | 337 |  |
| Total pneumococcal conjugate vaccine | | | | | 641 (730 minus 89) | |
| Rotavirus |  |  |  |  |  |  |
| Dose 1 | 2 months | 6 weeks | - | 3 months (90-93) | 156^e^ |  |
| Dose 2 | 4 months | 10 weeks | 4 weeks | 5 months (151-154) | 95^e^ |  |
| Total rotavirus | | | | | 156 (245 minus 89) | |
| Measles-containing vaccine^f^ |  |  |  |  |  |  |
| Dose 1 | 12 months | 12 months | - | 13 months (394-397) | 337 |  |
| Dose 2 | 18 months | 12 months + 4 weeks | 4 weeks | 19 months (578-581) | 153 |  |
| Total measles-containing vaccine | | | | | 337 (730 minus 393) | |

^a^ Number of days after which the recommended age of vaccination ends according to the date of birth (i.e. the day before reaching 3, 5, 13 and 19 months of age). This number may vary for each child.

**^b^** Maximum number of days that a child is considered overdue if not vaccinated before reaching 24 months of age. This number may vary for each child.

**^c^** Some children received the Hepatitis B vaccine at 0 (birth), 1 and 6 months of age. For these children, the minimum age and minimum intervals between doses differed, but the same recommended ages for routine vaccination were used to determine delays. Only 2 doses are required if administered ≥ 12 months of age, with a minimum interval of 5 months between doses.

**^d^** Only 2 doses are required if administered ≥ 12 months of age, with a minimum interval of 5 months between doses.

^e^ Delay count for rotavirus vaccine assessed up to 8 months of age (242-245 days). All doses were considered invalid if the first dose was administered after 20 weeks. Any dose given after 242-245 days was deemed invalid.

^f^ Measles-containing vaccine: all vaccines containing measles antigen (e.g. MMR; MMR±V)

**Supplementary Table 2** Yearly vaccination coverage (%) from 2019 to 2023 for routine immunization up to 24 months among Quebec children

| **Vaccine antigen** | **Evaluation age** | **2019** | | **2020** | | **2021** | | **2022** | | **2023** | | **P-trend** ^b^ |
| --- | --- | --- | --- | --- | --- | --- | --- | --- | --- | --- | --- | --- |
|  |  | **n^a^ (%)** | **95% CI** | **n^a^ (%)** | **95% CI** | **n^a^ (%)** | **95%CI** | **n^a^ (%)** | **95% CI** | **n^a^ (%)** | **95% CI** |  |
| **Diphtheria, tetanus, acellular pertussis-containing vaccine^c^** | 3 months | 80 248  (87.1) | 86.9, 87.3 | 76 044 (85.9) | 85.7, 86.1 | 77 428 (86.6) | 86.4, 86.8 | 75 644 (87.8) | 87.6, 88.0 | 70 911 (88.7) | 88.5, 88.9 | <.0001 |
|  | 5 months | 74 228 (80.3) | 80.0, 80.5 | 71 638 (79.8) | 79.5, 80.0 | 71 858 (81.5) | 81.2, 81.7 | 71 796 (81.9) | 81.7, 82.2 | 66 972 (82.6) | 82.3, 82.8 | <.0001 |
| **Hepatitis B**^d^ | 3 months | 79 717 (86.5) | 86.3, 86.7 | 75 661 (85.5) | 85.2, 85.7 | 77 092 (86.2) | 86.0, 86.4 | 75 403 (87.5) | 87.3, 87.7 | 70 694 (88.5) | 88.2, 88.7 | <.0001 |
|  | 5 months | 73 411 (79.4) | 79.1, 79.7 | 71 276 (79.4) | 79.1, 79.6 | 71 524 (81.1) | 80.8, 81.3 | 71 561 (81.7) | 81.4, 81.9 | 66 810 (82.4) | 82.1, 82.6 | <.0001 |
|  | 19 months | 56 145 (60.1) | 59.8, 60.4 | 51 175 (55.2) | 54.8, 55.5 | 55 717 (61.4) | 61.1, 61.7 | 51 971 (59.7) | 59.4, 60.0 | 53 434 (59.5) | 59.1, 59.8 | <.0001 |
| **Pneumococcal conjugate vaccine** | 3 months | 79 362 (86.1) | 85.9, 86.3 | 75 595 (85.4) | 85.2, 85.6 | 77 093 (86.2) | 86.0, 86.4 | 75 349 (87.5) | 87.2, 87.7 | 70 669 (88.4) | 88.2, 88.6 | <.0001 |
|  | 5 months | 73 068 (79.0) | 78.8, 79.3 | 71 085 (79.1) | 78.9, 79.4 | 71 417 (81.0) | 80.7, 81.2 | 71 436 (81.5) | 81.3, 81.8 | 66 672 (81.5) | 81.3, 81.8 | <.0001 |
|  | 13 months | 64 221 (69.0) | 68.7, 69.3 | 63 015 (68.6) | 68.3, 68.9 | 60 462 (69.4) | 69.1, 69.7 | 61 897 (68.2) | 67.9, 68.5 | 58 888 (69.4) | 69.1, 69.7 | 0.6253 |
|  | 15 months | 72 112 (77.5) | 77.3, 77.8 | 70 865 (77.1) | 76.8, 77.4 | 67 158 (77.1) | 76.8, 77.4 | 70 589 (77.8) | 77.5, 78.0 | 67 407 (79.4) | 79.2, 79.7 | <.0001 |
| **Rotavirus vaccine** | 3 months | 74 361 (80.7) | 80.4, 80.9 | 71 584 (80.9) | 80.6, 81.1 | 73 282 (81.9) | 81.7, 82.2 | 72 045 (83.6) | 83.4, 83.9 | 67 949 (85.0) | 84.8, 85.3 | <.0001 |
|  | 5 months | 67 407 (72.9) | 72.6, 73.2 | 66 834 (74.4) | 74.1, 74.7 | 67 344 (76.4) | 76.1, 76.6 | 67 853 (77.4) | 77.1, 77.7 | 63 663 (78.5) | 78.2, 78.8 | <.0001 |
| **Measles-containing vaccine^e^** | 13 months | 66 180 (71.1) | 70.9, 71.4 | 64 215 (69.9) | 69.6, 70.2 | 61 638 (70.8) | 70.5, 71.1 | 62 894 (69.3) | 69.0, 69.6 | 59 705 (70.3) | 70.0, 70.7 | <.0001 |
|  | 15 months | 74 717 (80.3) | 80.1, 80.6 | 72 574 (79.0) | 78.7, 79.2 | 68 717 (78.9) | 78.6, 79.2 | 72 041 (79.4) | 79.1, 79.6 | 68 587 (80.8) | 80.5, 81.1 | 0.0046 |
|  | 19 months | 56 956 (61.0) | 60.7, 61.3 | 52 007 (56.1) | 55.7, 56.4 | 56 324 (62.0) | 61.7, 62.4 | 52 632 (60.5) | 60.1, 60.8 | 53 881 (60.0) | 59.6, 60.3 | <.0001 |
|  | 24 months | 70 375 (75.4) | 75.1, 75.6 | 68 059 (73.4) | 73.1, 73.6 | 67 518 (74.4) | 74.1, 74.7 | 64 930 (74.6) | 74.3, 74.9 | 69 139 (76.9) | 76.7, 77.2 | <.0001 |

CI: Confidence intervals.

^a^ n: numerators used to calculate vaccination coverage. Denominators are presented in Supplementary Table 1.

^b^ The Cochran-Armitage test for trend was used.

^c^ All vaccines containing diphtheria, tetanus, acellular pertussis antigens (e.g. DTaP-HB-IPV, DTaP-IPV-Hib)

^d^ We presented hepatitis B vaccine separately from the DTaP-containing vaccine, as some infants received hepatitis B vaccine at birth and the vaccine used at the 18-month vaccination visit differed between cohorts included in this study (DTaP-HB-IPV-Hib vaccine for children born before June 2019 and the combined hepatitis A and hepatitis B vaccine for those born on or after 2019).

^e^ All vaccines containing measles antigen (e.g. MMR, MMRV).

**Supplementary Table 3** Yearly vaccination coverage (%) from 2019 to 2023 for routine immunization up to 24 months among Quebec children, excluding the two northern health regions of Quebec

| **Vaccine antigen** | **Evaluation age** | **2019** | | **2020** | | **2021** | | **2022** | | **2023** | | **P-trend** ^b^ |
| --- | --- | --- | --- | --- | --- | --- | --- | --- | --- | --- | --- | --- |
|  |  | **n^a^ (%)** | **95% CI** | **n^a^ (%)** | **95% CI** | **n^a^ (%)** | **95%CI** | **n^a^ (%)** | **95% CI** | **n^a^ (%)** | **95% CI** |  |
| **Diphtheria, tetanus, acellular pertussis-containing vaccine^c^** | 3 months | 79 707  (87.0) | 86.8, 87.2 | 75 548 (85.9) | 85.7, 86.1 | 76 885 (86.6) | 86.4, 86.8 | 75 147 (87.8) | 87.6, 88.1 | 70 570 (88.8) | 88.6, 89.0 | <.0001 |
|  | 5 months | 73 754 (80.3) | 80.0, 80.5 | 71 242 (79.8) | 79.6, 80.1 | 71 415 (81.6) | 81.3, 81.8 | 71 411 (82.1) | 81.8, 82.3 | 66 688 (82.7) | 82.5, 83.0 | <.0001 |
| **Hepatitis B**^d^ | 3 months | 79 177 (86.4) | 86.2, 86.7 | 75 166 (85.5) | 85.2, 85.7 | 76 548 (86.2) | 86.0, 86.4 | 74 907 (87.6) | 87.3, 87.8 | 70 342 (88.5) | 88.3, 88.7 | <.0001 |
|  | 5 months | 72 937 (79.4) | 79.1, 79.6 | 70 880 (79.4) | 79.1, 79.7 | 71 082 (81.2) | 80.9, 81.4 | 71 176 (81.8) | 81.5, 82.0 | 66 519 (82.5) | 82.2, 82.8 | <.0001 |
|  | 19 months | 55 751 (60.1) | 59.8, 60.5 | 50 849 (55.2) | 54.9, 55.5 | 55 433 (61.5) | 61.2, 61.8 | 51 713 (59.9) | 59.6, 60.3 | 53 177 (59.7) | 59.3, 60.0 | <.0001 |
| **Pneumococcal conjugate vaccine** | 3 months | 78 814 (86.0) | 85.8, 86.3 | 75 097 (85.4) | 85.1, 85.6 | 76 549 (86.2) | 86.0, 86.4 | 74 854 (87.5) | 87.3, 87.7 | 70 330 (88.5) | 88.3, 88.7 | <.0001 |
|  | 5 months | 72 586 (79.0) | 78.7, 79.2 | 70 684 (79.2) | 78.9, 79.5 | 70 974 (81.0) | 80.8, 81.3 | 71 050 (81.6) | 81.4, 81.9 | 66 391 (82.3) | 82.1, 82.6 | <.0001 |
|  | 13 months | 63 831 (69.0) | 68.7, 69.3 | 62 642 (68.6) | 68.3, 68.9 | 60 139 (69.5) | 69.2, 69.8 | 61 588 (68.3) | 68.0, 68.6 | 58 589 (69.5) | 69.2, 69.8 | 0.1599 |
|  | 15 months | 71 639 (77.5) | 77.2, 77.7 | 70 412 (77.1) | 76.8, 77.4 | 66 738 (77.1) | 76.8, 77.4 | 70 118 (77.8) | 77.5, 78.1 | 67 023 (79.5) | 79.2, 79.8 | <.0001 |
| **Rotavirus vaccine** | 3 months | 73 808 (80.6) | 80.3, 80.8 | 71 089 (80.8) | 80.6, 81.1 | 72 736 (81.9) | 81.7, 82.2 | 71 550 (83.6) | 83.4, 83.9 | 67 609 (85.1) | 84.8, 85.3 | <.0001 |
|  | 5 months | 66 928 (72.8) | 72.5, 73.1 | 66 445 (74.4) | 74.2, 74.7 | 66 901 (76.4) | 76.1, 76.7 | 67 465 (77.5) | 77.2, 77.8 | 63 384 (78.6) | 78.3, 78.9 | <.0001 |
| **Measles-containing vaccine^e^** | 13 months | 65 778 (71.1) | 70.8, 71.4 | 63 833 (69.9) | 69.6, 70.2 | 61 317 (70.9) | 70.6, 71.2 | 62 578 (69.4) | 69.1, 69.7 | 59 400 (70.5) | 70.2, 70.8 | 0.0002 |
|  | 15 months | 74 229 (80.3) | 80.0, 80.5 | 72 111 (79.0) | 78.7, 79.2 | 68 286 (78.9) | 78.7, 79.2 | 71 560 (79.4) | 79.1, 79.7 | 68 186 (80.9) | 80.6, 81.2 | 0.0003 |
|  | 19 months | 56 494 (60.9) | 60.6, 61.3 | 51 613 (56.0) | 55.7, 56.3 | 55 980 (62.1) | 61.8, 62.4 | 52 337 (60.7) | 60.3, 61.0 | 53 554 (60.1) | 59.8, 60.4 | <.0001 |
|  | 24 months | 69 721 (75.2) | 75.0, 75.5 | 67 481 (73.3) | 73.0, 73.6 | 66 982 (74.4) | 74.1, 74.6 | 64 346 (74.6) | 74.3, 74.9 | 68 509 (77.0) | 76.7, 77.3 | <.0001 |

CI: Confidence intervals.

^a^ n: numerators used to calculate vaccination coverage. Denominators are presented in Supplementary Table 1.

^b^ The Cochran-Armitage test for trend was used.

^c^ All vaccines containing diphtheria, tetanus, acellular pertussis antigens (e.g. DTaP-HB-IPV, DTaP-IPV-Hib)

^d^ We presented hepatitis B vaccine separately from the DTaP-containing vaccine, as some infants received hepatitis B vaccine at birth and the vaccine used at the 18-month vaccination visit differed between cohorts included in this study (DTaP-HB-IPV-Hib vaccine for children born before June 2019 and the combined hepatitis A and hepatitis B vaccine for those born on or after 2019).

^e^ All vaccines containing measles antigen (e.g. MMR, MMRV).

**Supplementary Table 4** Days undervaccinated for vaccines included in the analysis among Quebec children born from June 2017 to May 2023, excluding the two northern health regions of Quebec, 2019-2023

|  | Birth Cohort (No. combined = 450 345)^a^ | | | | |
| --- | --- | --- | --- | --- | --- |
|  | Children born from June 2017 to May 2018 | Children born from June 2018 to May 2019 | Children born from June 2019 to May 2020 | Children born from June 2020 to May 2021 | Children born from June 2021 to May 2022 |
|  | 18-month visit in 2019 | 18-month visit in 2020 | 18-month visit in 2021 | 18-month visit in 2022 | 18-month visit in 2023 |
| Children included in the analysis | 92 697 | 92 116 | 90 102 | 86 293 | 89 137 |
| No. days undervaccinated, Mean (SD) | | | | | |
| Pneumococcal conjugate vaccine | | | | | |
| Children with vaccine delay^b^ , n (%) | 35 299 (38.1) | 33 436 (36.3) | 30 890 (34.3) | 30 109 (34.9) | 30 979 (34.8) |
| Dose 1 | 77.8 (0.7) | 69.0 (0.6) | 66.6 (0.6) | 68.6 (0.7) | 58.8 (0.6) |
| Dose 2 | 83.0 (0.6) | 74.3 (0.6) | 75.0 (0.6) | 75.5 (0.6) | 67.2 (0.6) |
| Dose 3 | 73.9 (0.4) | 69.7 (0.4) | 69.0 (0.4) | 70.3 (0.4) | 64.3 (0.4) |
| Total^c^ | 82.2 (0.5) | 77.6 (0.5) | 78.1 (0.5) | 78.7 (0.5) | 73.2 (0.5) |
| Hepatitis B | | | | | |
| Children with vaccine delay^b^ n (%) | 41 065 (44.3) | 44 579 (48.4) | 38 231 (42.4) | 36 880 (42.7) | 38 691(43.4) |
| Dose 1 | 77.5 (0.7) | 69.7 (0.6) | 68.4 (0.6) | 70.4 (0.7) | 60.2 (0.6) |
| Dose 2 | 85.5 (0.7) | 77.9 (0.6) | 79.0 (0.6) | 79.4 (0.7) | 70.7 (0.6) |
| Dose 3 | 45.6 (0.2) | 50.5 (0.2) | 45.7 (0.2) | 46.2 (0.2) | 43.8 (0.2) |
| Total^c^ | 102.4 (0.6) | 102.4 (0.6) | 98.6 (0.6) | 99.3 (0.6) | 91.8 (0.6) |
| Rotavirus | | | | | |
| Children with vaccine delay^b^ n (%) | 28 288 (30.5) | 26 071 (28.3) | 23 701 (26.3) | 21 212 (24.6) | 21 006 (23.6) |
| Dose 1 | 30.6 (0.2) | 27.8 (0.2) | 25.0 (0.2) | 25.2 (0.2) | 21.9 (0.2) |
| Dose 2 | 22.5 (0.1) | 20.7 (0.1) | 19.4 (0.1) | 18.8 (0.1) | 17.2 (0.1) |
| Total^c,d^ | 35.3 (0.2) | 32.4 (0.2) | 29.9 (0.2) | 29.4 (0.2) | 26.5 (0.2) |
| Measles-containing vaccine^e^ | | | | | |
| Children with vaccine delay^b^ n (%) | 41 129 (44.4) | 45 322 (49.2) | 38 143 (42.3) | 38 712 (44.9) | 40 978 (46.0) |
| Dose 1 | 60.3 (0.4) | 59.1 (0.4) | 60.6 (0.4) | 62.2 (0.4) | 57.5 (0.4) |
| Dose 2 | 43.2 (0.2) | 48.2 (0.2) | 43.9 (0.2) | 44.3 (0.2) | 42.4 (0.2) |
| Total^c^ | 80.8 (0.4) | 84.6 (0.4) | 80.7 (0.4) | 82.3 (0.4) | 78.1 (0.4) |
| All vaccines^f^ | 159.2 (0.8) | 154.5 (0.7) | 144.5 (0.7) | 144.5 (0.8) | 133.3 (0.7) |
| All vaccines (without rotavirus) | 148.5 (0.8) | 144.3 (0.7) | 136.6 (0.7) | 137.0 (0.8) | 126.7 (0.7) |
| Average no of days undervaccinated^g^ | 75.2 (0.2) | 74.3 (0.2) | 71.8 (0.2) | 72.4 (0.2) | 67.4 (0.2) |
| Average no of days undervaccinated (without rotavirus) | 66.4 (0.3) | 66.1 (0.3) | 64.4 (0.3) | 65.1 (0.3) | 60.8 (0.3) |

SD: Standard deviation. PCV: pneumococcal conjugate vaccine; HB: hepatitis B vaccine; Rota: rotavirus vaccine.

^a^ Cohorts were selected based on vaccination visits scheduled each year from 2019 to 2023. Only the cohorts selected for the 18-month vaccination visit had complete follow-up to 24 months and were included in the analysis of vaccination delays.

^b^ At least one dose that is delayed.

^c^ Days undervaccinated for each specific vaccine were calculated by summing all days during which the child was undervaccinated for at least one dose of that vaccine. Overlapping days are only counted once. For instance, if a child is considered undervaccinated for dose 1 of the rota vaccine on days 90 to 180 (received the dose at 6 months) and for dose 2 on days 151 to 210 (received the dose at 7 months), the overlapping days from 151 to 180 were only counted once and the child will be considered undervaccinated for a total of 121 days (from 90 days to 210 days).

^d^ Delay count for rotavirus vaccine assessed up to 8 months of age (242-245 days). All doses were considered invalid if the first dose was administered after 20 weeks. Any dose given after 242-245 days was deemed invalid.

^e^ Measles-containing vaccine: all vaccines containing measles antigen (e.g. MMR, MMRV)

^f^ Days undervaccinated for all vaccines were calculated by summing all days during which the child was undervaccinated for at least one dose of any vaccines included in the analysis. Overlapping days are only counted once.

^g^ Calculated as the total number of days undervaccinated summed across all vaccine series by the number of vaccines recommended (no=4)

**Supplementary Figure 1**Cumulative proportion of Quebec children vaccinated by month in 2019 and 2023 for the measles-containing vaccine (doses 1 and 2) (A), as well as for the pneumococcal conjugate vaccine, the hepatitis B vaccine and the rotavirus vaccine (dose 1) (B)


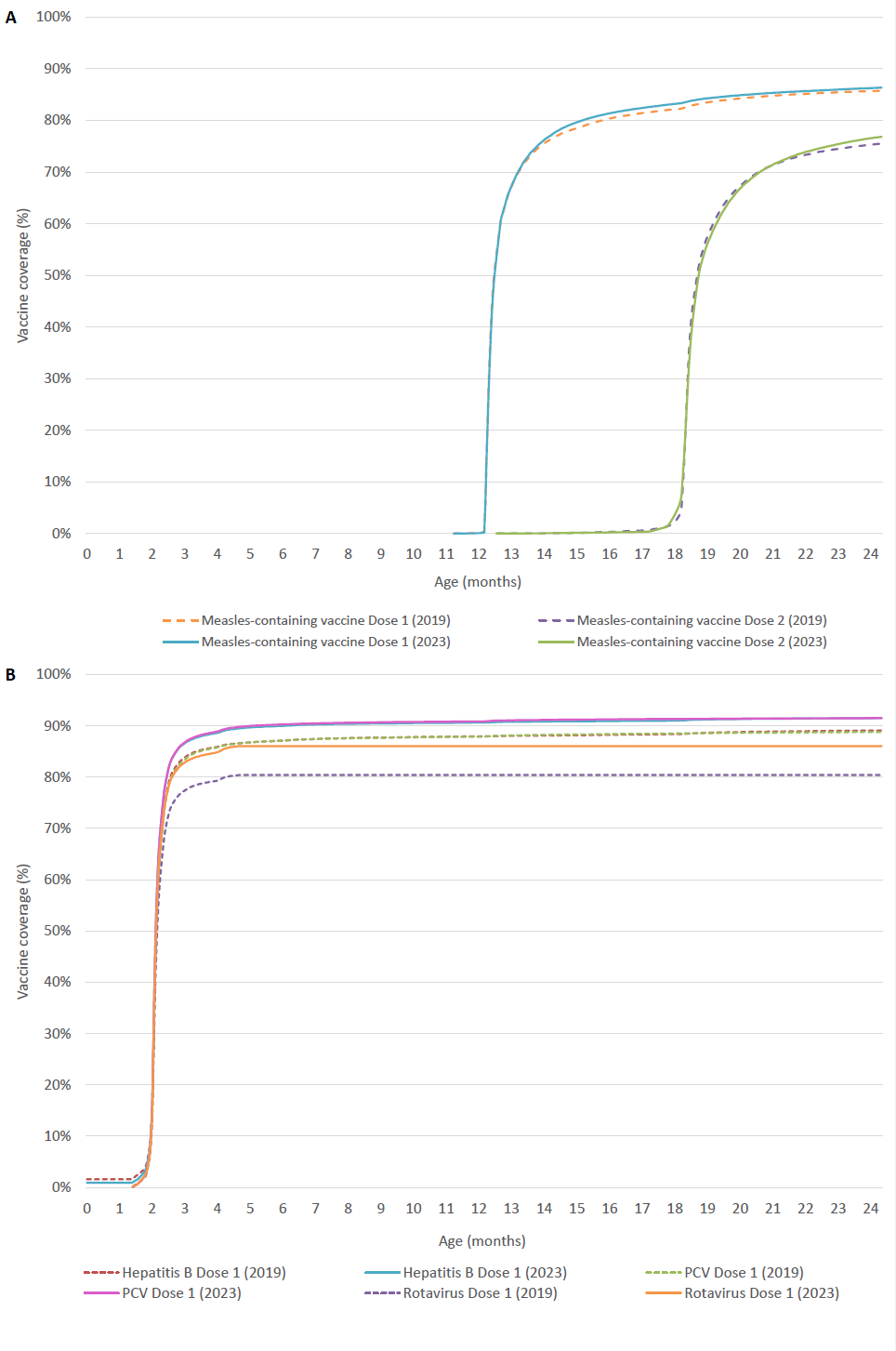


Measles-containing vaccine: all vaccines containing measles antigen (e.g. MMR, MMRV) PCV: pneumococcal conjugate vaccine.
